# Leveraging pleiotropic association using sparse group variable selection in genomics data

**DOI:** 10.1186/s12874-021-01491-8

**Published:** 2022-01-07

**Authors:** Matthew Sutton, Pierre-Emmanuel Sugier, Therese Truong, Benoit Liquet

**Affiliations:** 1 Queensland University of Technology Centre for Data Science, Brisbane, Australia; 2 Laboratoire De Mathématiques et de leurs Applications de PAU E2S UPPA, CNRS, Pau, France; 3 University Paris-Saclay, UVSQ, Inserm, Gustave Roussy, CESP, Team “Exposome and Heredity”, Villejuif, France; 4 Department of Mathematics and Statistics, Macquarie University, Sydney, Australia

**Keywords:** Genetic epidemiology, High dimensional data, Lasso penalization, Oncology, Pathway analysis, Pleiotropy, Sparse methods, Variable selection

## Abstract

**Background:**

Genome-wide association studies (GWAS) have identified genetic variants associated with multiple complex diseases. We can leverage this phenomenon, known as pleiotropy, to integrate multiple data sources in a joint analysis. Often integrating additional information such as gene pathway knowledge can improve statistical efficiency and biological interpretation. In this article, we propose statistical methods which incorporate both gene pathway and pleiotropy knowledge to increase statistical power and identify important risk variants affecting multiple traits.

**Methods:**

We propose novel feature selection methods for the group variable selection in multi-task regression problem. We develop penalised likelihood methods exploiting different penalties to induce structured sparsity at a gene (or pathway) and SNP level across all studies. We implement an alternating direction method of multipliers (ADMM) algorithm for our penalised regression methods. The performance of our approaches are compared to a subset based meta analysis approach on simulated data sets. A bootstrap sampling strategy is provided to explore the stability of the penalised methods.

**Results:**

Our methods are applied to identify potential pleiotropy in an application considering the joint analysis of thyroid and breast cancers. The methods were able to detect eleven potential pleiotropic SNPs and six pathways. A simulation study found that our method was able to detect more true signals than a popular competing method while retaining a similar false discovery rate.

**Conclusion:**

We developed feature selection methods for jointly analysing multiple logistic regression tasks where prior grouping knowledge is available. Our method performed well on both simulation studies and when applied to a real data analysis of multiple cancers.

## Background

In recent years, genome-wide association studies (GWAS) have identified genetic variants associated to multiple traits. The phenomenon where one genetic loci affects multiple different phenotypes is called pleiotropy [1]. A comprehensive overview of the genetic architecture in complex traits from GWAS datasets reported that 31% of single nucleotide polymorphisms (SNPs) and 63% of genes were pleiotropic [2]. Identification of these pleiotropic effects may help to understand the shared etiology among complex diseases by highlighting common biological pathway. As a consequence of the active interest in pleiotropy, there are now a number of statistical tools which identify pleiotropic signal [3–7]. See [8] for a survey.

Amongst these methods one of the most popular for practitioners is a subset based meta-analysis *ASSET* [9]. This method exhaustively explores subsets of the phenotypes for the detection of associated variants, regardless of the direction of the effects. The method returns a p-value that can be used to determine the significance of potential pleiotropic effects. ASSET is a popular method that has received frequent use in the analysis of multiple diseases [10, 11].

Among existing methods, mixture model methods have received attention for modelling pleiotropic associations [12]. These methods partition the SNPs into those that are associated to multiple traits (pleiotropic association), associated with a single trait or not associated to a trait. These methods have recently been extended to integrate functional annotations to improve the power in pleiotropic mapping [7]. Chung et al. [13] proposed a method for genetic analysis incorporating pleiotropy and annotation (GPA). These methods were later extended by Liu et al. [14] who made use of extended mixture models to allow for the incorporation of gene set analysis.

In this article, we propose novel methods which model pleiotropy for genomics data in the case of independent datasets. Our methods are developed to model pleiotropic correlation amongst jointly analysed traits and account for the gene structure information contained in the data. Integrating additional information such as gene pathway knowledge offers the potential to improve statistical efficiency. Our statistical approach exploits both gene (or pathway) and pleiotropy knowledge to increase the statistical power of identifying risk variants shared by multiple diseases. We conduct simulation studies to evaluate the performance of our method.

Our method can be motivated in a multi-task framework [15]. In our context, each genetic dataset would correspond to a different learning task. A common approach to this setting is to assume that only a few common important genes are shared across the *K* studies. In particular, we make note of Lounici et al. [16] who consider using the Group Lasso as a candidate estimation method for multi-task estimation in linear regression models. These ideas have since been extended by Wang et al. [17] who propose a penalised likelihood approach for multi-task regression which can incorporate group structure. Their method is proposed for the case when the response is a set of continuous responses. We extend their penalisation methods to account for a binary response variable.

We are motivated by an application of our methods to the analysis of pleiotropy between thyroid and breast cancers. Thyroid and breast cancers share some similarities: both are more frequent in women, are influenced by reproductive factors and are hormonally-mediated. Moreover, individuals diagnosed with breast cancer are more likely to develop thyroid cancer as a secondary malignancy than patient diagnosed with other cancer types [18]. These associations do not seem to be explained totally by surveillance bias or treatment effect, but rather suggests common lifestyle risk factors (such as reproductive factors, diet or obesity) or shared genetic susceptibility that still need to be explored. By jointly analysing the genetic relationships of breast and thyroid cancers, we aim to understand the nature of the association between the two cancers and identify potential common biological mechanisms.

The remainder of this article is organised as follows. In methods section, we describe the core model, algorithm and inference method. A stability exploration based on a bootstrap approach is provided. Our method is tested in a simulation study where we compare the joint penalised likelihood approach to state-of-the-art variable selection strategies to investigate pleiotropy. The results section present the results of the simulation study and the pleiotropy investigation on thyroid and breast cancers. The final section concludes with a discussion of the methods and potential extensions of the work.

## Methods

### Modelling sparse and grouped associations in many independent datasets

Suppose we have data from *K* independent datasets, $\mathcal {D} = \mathcal {D}_{1} \cup \mathcal {D}_{2} \cup \cdots \cup \mathcal {D}_{K}$, where $\phantom {\dot {i}\!}\mathcal {D}_{k} = (\{y_{1k}, x_{1k}\},\dots,\{y_{n_{k} k}, x_{n_{k} k}\})$ and dataset contain *n*_1_,…,*n*_*K*_ samples respectively. The response variable *y*_*ik*_∈{0,1} is the binary phenotype of the *i*th individual of the *k*th study and $x_{{ik}} \in \mathbb {R}^{p}$ is the vector with corresponding *p* variables of the *i*th individual of the *k*th study. These data are assumed to come from a logistic regression model where

$$\begin{array}{*{20}l} p\left(Y_{{ik}}=y_{{ik}}|X_{{ik}}=x_{{ik}}\right) = \frac{\exp\left(y_{{ik}}x_{{ik}}^{T} \beta_{\cdot k}\right)}{1 + \exp\left(x_{{ik}}^{T} \beta_{\cdot k}\right)}  \end{array} $$

for *k*=1,…,*K*, where $\beta _{\cdot k} \in \mathbb {R}^{p}$ denotes the regression coefficients for the *k*th study. To simplify further notation, let $\beta _{j \cdot } \in \mathbb {R}^{K}, j=1,\dots,p$ denote the vector of the *K* regression coefficients corresponding to the *j*th SNP over the *K* datasets. We let *β*_*jk*_ denote the regression coefficient for the *j*th SNP of the *k*th study. We assume that the set of SNPs can be partitioned into *G* groups where each SNP belongs to a single unique group. Let *π*_*g*_,*g*=1,…,*G* denote the set of SNPs contained in the *g*th group and *n*_*g*_ be the number of SNPs in group *g*. Finally, we let the matrix of all regression coefficients be denoted by ***B***=(*β*_·1_,…,*β*_·*K*_). Since the *K* multiple studies were observed independently, the negative log likelihood for the combined datasets has the generic expression:

1 $$\begin{array}{*{20}l}  \ell(\boldsymbol{B};\mathcal{D}) = -\sum_{k=1}^{K} \sum_{i=1}^{n_{k}} \left(y_{i} x_{{ik}}^{T} \beta_{\cdot k} - \log(1 + e^{x_{{ik}}^{T} \beta_{\cdot k}})\right) \end{array} $$

where $\ell (\boldsymbol {B};\mathcal {D})$ denotes the negative log likelihood for the observed data $\mathcal {D}$.

### Sparse group multi-Task method

Our *Sparse Group Multi-Task* (SGMT) approach is based on penalised likelihood maximisation. Using the likelihood form for independent datasets (1), we propose the penalised likelihood estimate

2 $$ \begin{aligned}  \hat{\boldsymbol{B}} &= \underset{\boldsymbol{B} \in \mathbb{R}^{p \times K}}{\text{argmin}} \left\{ \ell(\boldsymbol{B};\mathcal{D}) \quad + \quad \lambda(1-\alpha) \| \boldsymbol{B} \|_{G_{2,1}} \quad + \quad \lambda\alpha \| \boldsymbol{B} \|_{l_{2,1}} \right\} \\ &\text{where} \| \boldsymbol{B} \|_{G_{2,1}} = \sum_{g=1}^{G} \sqrt{n_{g}} \sqrt{\sum_{i \in \pi_{g}} \sum_{k=1}^{K} \beta_{{ik}}^{2}} \\ &\text{and }\| \boldsymbol{B} \|_{l_{2,1}} = \sum_{i=1}^{p} \| \beta_{i \cdot} \|_{2} = \sum_{i=1}^{p} \sqrt{\sum_{k=1}^{K} \beta_{{ik}}^{2}} \end{aligned}  $$

where *λ*≥0 and *α*∈[0,1] are regularisation parameters weighting a *G*_2,1_-norm penalty $\| \boldsymbol {B} \|_{G_{2,1}}$ and *l*_2,1_-norm penalty $\| \boldsymbol {B} \|_{l_{2,1}}$. The parameter *λ* controls an overall amount of penalisation, while *α* determines how much penalisation is used for each penalty. The *G*_2,1_-norm [19] fixes the group structure across studies and encourage sparsity at group-level. As important groups may contain irrelevant SNPs we desire a method which is able to select variables within a group. This is handled by the *l*_2,1_-norm which allows for more structured sparsity. The penalisation matches the penalisation proposed in Wang et al. [19] but differs due to the logistic likelihood.

Equation (2) enables us to define three models:

1. *Grouped multi-task penalised model* (GMT) by fixing *α*=0.
2. *Sparse multi-task penalised model* (SMT) by fixing *α*=1.
3. *Sparse Grouped multi-task penalised model* (SGMT) with 1>*α*>0.

### Optimization algorithm

We propose to fit this model (Eq. 2) using the alternating direction method of multipliers (ADMM) algorithm [20]. To simplify the notation we define *λ*_1_=(1−*α*)*λ* and *λ*_2_=*λα*. The ADMM formulation of our optimisation problem is given by

$$\begin{aligned} \underset{\boldsymbol{B, Z}}{\text{min}} \left\{ \ell(\boldsymbol{B};\mathcal{D}) \quad + \quad \lambda_{1} \| \boldsymbol{Z} \|_{G_{2,1}} \quad \!+ \quad \lambda_{2} \| \boldsymbol{Z} \|_{l_{2,1}} \right\} \quad \text{subject to}\ \boldsymbol{Z} \,=\, \boldsymbol{B}. \end{aligned} $$

where $\boldsymbol {Z} \in \mathbb {R}^{p \times K}$. The augmented Lagrangian introduces auxiliary variable ***U*** with Lagrange multiplier *ρ* and is given by the following:

$$\begin{array}{*{20}l}  \mathcal{L}_{\rho} (\boldsymbol{B}, \boldsymbol{Z}, \mathbf{U}) =&\ \ell(\boldsymbol{B};\mathcal{D}) \quad + \quad \lambda_{1} \| \boldsymbol{Z} \|_{G_{2,1}} \quad + \quad \lambda_{2} \| \boldsymbol{Z} \|_{l_{2,1}} \\ &+ \quad \frac{\rho}{2} \| \boldsymbol{B} - \boldsymbol{Z} + \boldsymbol{U} \|_{F}^{2} \quad + \quad \frac{\rho}{2} \| \boldsymbol{U} \|_{F}^{2} \end{array} $$

The ADMM algorithm makes the following set of updates:

$$\begin{array}{*{20}l} \boldsymbol{B}^{t+1} &= \underset{\boldsymbol{B} \in \mathbb{R}^{p \times K}}{\text{argmin}} \quad \mathcal{L}_{\rho} (\boldsymbol{B}, \boldsymbol{Z}^{(t)}, \boldsymbol{U}^{(t)}) \\ \boldsymbol{Z}^{t+1} &= \underset{\boldsymbol{Z} \in \mathbb{R}^{p \times K}}{\text{argmin}} \quad \mathcal{L}_{\rho} (\boldsymbol{B}^{(t+1)}, \boldsymbol{Z}, \boldsymbol{U}^{(t)}) \\ \boldsymbol{U}^{t+1} &= \boldsymbol{U}^{(t)} + \boldsymbol{B}^{(t+1)} - \boldsymbol{Z}^{(t+1)}. \end{array} $$

Each iterations of the algorithm consist of three sub-problems. In this case, we obtain an *l*_2_ regularisation logistic regression, a convex optimisation problem and a dual variable update (respectively):

$$\begin{aligned} \boldsymbol{B}^{t+1} =& \underset{\boldsymbol{B} \in \mathbb{R}^{p \times K}}{\text{argmin}} \quad \ell(\boldsymbol{B};\mathcal{D}) + \frac{\rho}{2} \| \boldsymbol{B} - \boldsymbol{Z}^{(t)} + \boldsymbol{U}^{(t)} \|_{F}^{2} \\ \boldsymbol{Z}^{t+1} =& \underset{\boldsymbol{Z} \in \mathbb{R}^{p \times K}}{\text{argmin}} \quad \frac{1}{2} \| \boldsymbol{B}^{(t+1)} + \boldsymbol{U}^{(t)} - \boldsymbol{Z} \|_{F}^{2} \quad + \frac{\lambda_{1}}{\rho} \| \boldsymbol{Z} \|_{G_{2,1}} \\&+ \frac{\lambda_{2}}{\rho} \| \boldsymbol{Z} \|_{l_{2,1}} \\ \boldsymbol{U}^{t+1} =& \boldsymbol{U}^{(t)} + \boldsymbol{B}^{(t+1)} - \boldsymbol{Z}^{(t+1)} \end{aligned} $$

The optimisation for the *l*_2_ regularised logistic regression is solved using the efficient Limited-memory Brouden-Fletcher-Golfarb-Shanno (L-BFGS) algorithm implemented in the RcppNumerical package. Let $[\boldsymbol {A}]_{(\pi _{g}, \cdot)}$ denote the rows of a matrix ***A*** corresponding to the SNP indices in *π*_*g*_. Following [21], the update ***Z***^(*t*+1)^ consists of the following two loops:

1. for *j*=1,…,*p*
  $$\begin{array}{*{20}l} [\boldsymbol{Z}^{(t+1)}]_{(j,\cdot)} = \mathcal{S}_{\lambda_{1}} ([\boldsymbol{B}^{(t+1)} + \boldsymbol{U}^{(t)}]_{(j,\cdot)}) \end{array} $$
2. for *g*=1,…,*G*
  $$\begin{array}{*{20}l} [\boldsymbol{Z}^{(t+1)}]_{(\pi_{g},\cdot)} = \mathcal{S}_{\lambda_{2}} ([\boldsymbol{Z}^{(t+1)}]_{(\pi_{g},\cdot)}) \end{array} $$
  where
  $$\begin{array}{*{20}l} \mathcal{S}_{\lambda} (\boldsymbol{A}) = \left\{\begin{array}{ll} \boldsymbol{0}, & \text{if }~ \|\boldsymbol{A}\|_{F} \leq \lambda \\ \frac{\|\boldsymbol{A}\|_{F} - \lambda}{\|\boldsymbol{A}\|_{F}}\boldsymbol{A}, & \text{otherwise.} \end{array}\right. \end{array} $$

### Calibration of tuning parameters

Tuning parameters *λ* and *α* are calibrated using a K-fold cross validation with deviance loss. We recommend repeated K-fold cross-validation to get more insight of the variability of the estimated deviance loss. An user friendly function is provided from our R package and an example can be found in the supplementary materials.

### Stability analysis

The different models (SMT, GMT and SGMT) are fitted using tuning parameters chosen by repeated K-fold cross-validation. Genes (or pathways) are then detected and *selected* as pleiotropic and others genes are *not selected*. These methods provide simultatious model fitting and selection. However, the challenge of inference for these sparse estimators is notoriously difficult. Recent work has developed theoretical results the sampling distribution of the Lasso estimator, allowing for p-value calculations. This allows for inference on the statistical strength of included variables. However, these results require technical theoretical development and can be complicated when using the adaptive lasso and more complex penalisation approaches. The stability of our proposed models are explored using a bootstrap strategy [22]. This non-parametric approach is commonly applied to provide inference on the stability of the selected variables in penalised methods [23, 24]. Bootstrapping for penalised methods has been studied theoretically [23, 25] and for practical use in GWAS analysis [26].

Resampling bootstrap is used in our approach where the different models are implemented on each bootstrap using tuning parameters selected from the original data. The frequency of the *selected genes* (or pathways) and *non selected genes* (pathways) over the *M* bootstrap samples quantifies the stability of the selected variables. We report both the variables selected and the selection rates for the application. More details on the bootstrapping procedures are given in the Results and Applications sections. Specifically, we estimate the probability of selection for each variable (or group of variables) with a given set of tuning parameters (*α*,*λ*) based on the proportion of times they are included on the bootstrapped fits. Commonly in GWAS data we are concerned with controlling the false discovery rate (FDR). One way to approach controlling this would be to specify a cut-off for the minimum probability of inclusion for each variable. In our simulation study we consider a strict control, only selecting variables that are included on every bootstrap. This conservative strategy would often be too drastic and for or application section we adopt another approach. This is to report the variables that had selection probabilities at least as high as the selection probabilities of the variables included on the full data fit. We state both the variables and their bootstrapped selection rates. More details on the bootstrapping procedures are given in the Results and Applications sections. An example demonstrating the Bootstrapping approaches is given in the supplementary material and can be reproduced from github.com/matt-sutton/SGMT.

### Adaptive weights

While penalised approaches allow for shrinkage of coefficients to zero, they come at the cost of possibly excessive shrinkage to non-zero coefficients. This has motivated a number of approaches that aim to reduce the effect of shrinkage on non-zero coefficients. One simple approach is the adaptive lasso [27]. The adaptive lasso approach takes the standard *ℓ*_1_ penalty of the lasso $\sum _{j=1}^{p}|\beta _{j}|$ and assigns weights to each coefficient $\sum _{j=1}^{p}w_{j}|\beta _{j}|$. Using an appropriate choice for the weights penalisation for non-zero coefficients can be reduced and these coefficients will suffer less shrinkage. A common choice for the weights is $w_{j} = 1/|\hat {\beta _{j}}|$ where $\hat {\beta _{j}}$ is the ordinary least squares estimate of the *j*th coefficient. Similar to the adaptive lasso we also allow a weighted version where the *G*_2,1_-norm and *ℓ*_2,1_-norm penalties are replaced by,

$$\sum_{g=1}^{G} \gamma_{g} \sqrt{\sum_{i \in \pi_{g}} \sum_{k=1}^{K} \beta_{{ik}}^{2}}, \qquad \text{ and} \quad \sum_{i=1}^{p}\kappa_{i} \sqrt{\sum_{k=1}^{K} \beta_{{ik}}^{2}} $$

respectively. Analogous to similar adaptive group and sparse-group lasso material in the literature [28, 29], the *G*_2,1_-norm weights *γ*_*g*_ are taken to be the inverse of the *G*_2,1_-norm of the OLS coefficients for *g*=1,...,*G*. Similarly we take the weights for the *ℓ*_2,1_-norm to be *κ*_*i*_ where *κ*_*i*_ is chosen as the inverse of the *ℓ*_2,1_-norm applied to the OLS coefficients for *i*=1,...,*p*. That is, we set the weights to be:

$$\gamma_{g} = \frac{1}{\sqrt{\sum_{i \in \pi_{g}} \sum_{k=1}^{K} \hat{\beta}_{{ik}}^{2}}}, \qquad \text{ and} \quad \kappa_{i} = \frac{1}{\sqrt{\sum_{k=1}^{K} \hat{\beta}_{{ik}}^{2}}}. $$

Alternative choices for the weighting function could also be considered and would be a topic of interesting further work. In addition one could also consider reducing the shrinkage effect by refitting the solution naively on the selected variables. While this topic has received some attention in penalised linear regression modelling [30] it has received less in logistic regression and is in general an open question.

### Simulation design

To assess the correctness and efficiency of our methods, we run simulations and compare the results with the well known frequentist approaches ASSET and GPA for detecting pleiotropic signal [9, 13]. In order to show the contribution of leveraging pleiotropy, we also run and compare the results of our novel approaches with the single-task group lasso (SGST), in which each trait is treated individually. The simulations and implementations of all methods have been carried out in R.

At each simulation, *K*=2 datasets are generated. We look at monitoring the efficiency of our methods for detecting effects across the multiple studies. In particular, we considered the effect of grouping information on the efficiency of the methods. The simulations were set to range from a simulation setting in which the grouping information was not as useful, the groups were almost entirely set to zero, to one where grouping information entirely determined the selected variables, i.e every variable in the group was active. The true effect size for any active variable was set to 0.8, and active variables in study 1 were all set positive, while those in study 2 alternated (allowing for same and different direction pleiotropic effects).

**Fig. 1:**
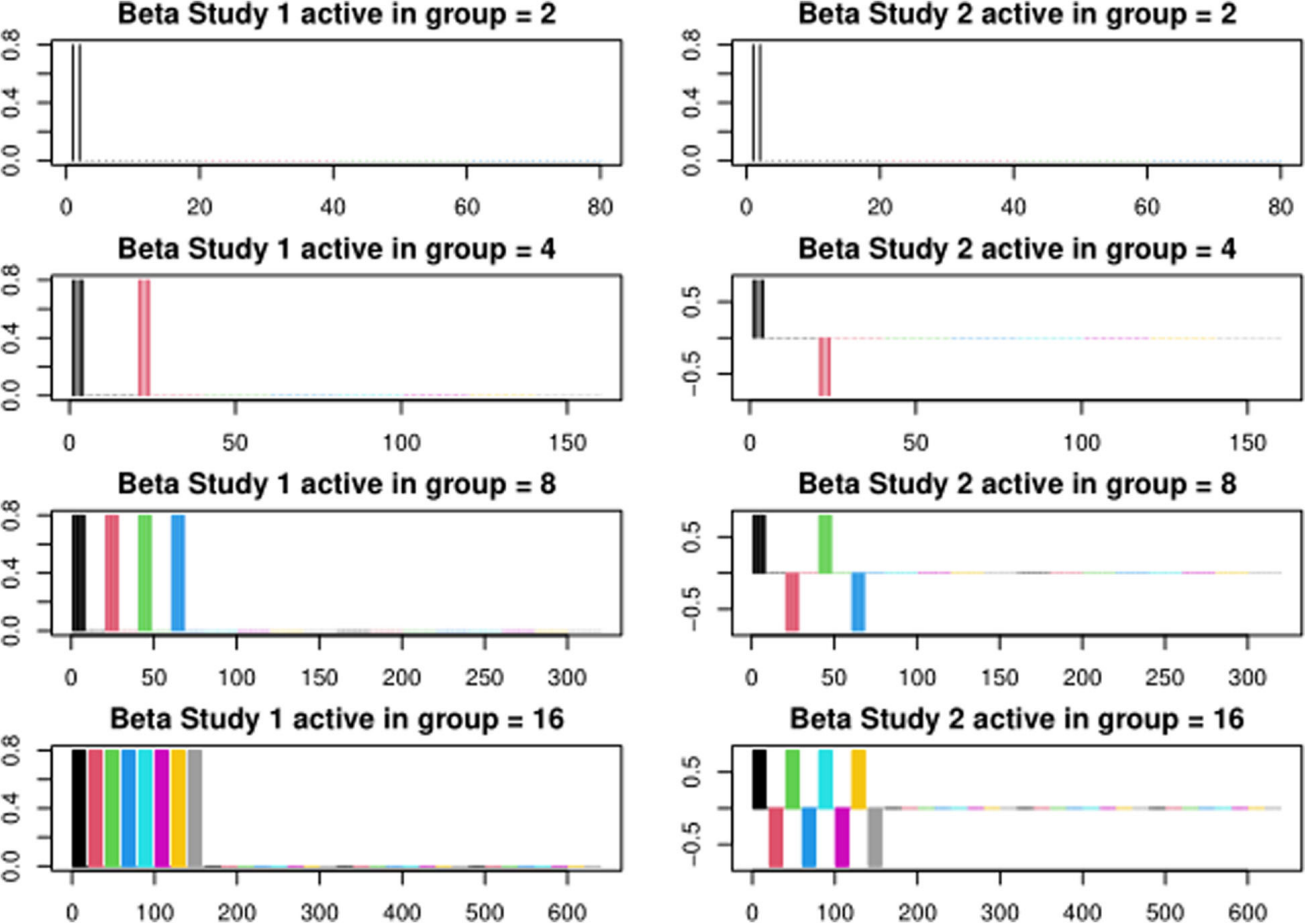
Each row in the figure corresponds to a simulated scenario. Colours correspond to groups, and the number active in a group refers to the number of non-zero variables *p*_*a*_ in a group consisting of 20 variables per study (so 40 variables over *K*=2 studies). The number of non-zero vs zero groups is (1/4,2/8,4/16 and 8/32)

We considered four simulation settings where we increased the number of active variables *p*_*a*_ within groups consisting of 20 variables. This was set to *p*_*a*_=2,4,8 and 16 active variables out of the 20 variables within each group. Each variable occurs in both of the *K*=2 studies so the total number of effects to be estimated within a group is 40. To offset the effect of having more active variables when *p*_*a*_ is larger, we increase the total number of variables in these simulations, considering *p*=80,160,320 and 640 variables (corresponding to 160, 320, 640 and 1280 estimated effects across the studies). Under this design, simulations should naturally favour sparse methods such as ASSET or SMT initially and GMT as the group structure becomes more relevant (higher *p*_*a*_ values). The simulation settings are given in more detail in Fig. 1. The total numbers of observations for the simulations were *n*=100,200,400 and 800, keeping the ratio *p*/*n* constant, with half of the observations in each study. The ratio of number of active groups to total number of groups was kept constant across the simulations so that the difficulty of group selection was consistent.

Once data are generated, our novel methods are applied and compared to the R implementation of ASSET using default parameter settings [9]. We want to compared these methods on their ability to recover the coefficients. We are not interested in the prediction accuracy. For each method, the true positive rate (TPR), the true negative rate (TNR), the *ℓ*_1_ error $\sum _{i=1}^{p} \sum _{k=1}^{K} | \beta _{{ik}} - \hat {\beta }_{{ik}}| $, the *ℓ*_2_ error $\sqrt {\sum _{i=1}^{p} \sum _{k=1}^{K} (\beta _{{ik}} - \hat {\beta }_{{ik}})^{2}}$ and the Matthews correlation coefficient (MCC) [31] are computed. The computing time is also collected. The simulations are replicated 100 times. An additional simulation exploring performance when *K*=3 is considered in the supplementary material. This additional simulation was taken as the simulation here for *K*=2 with an additional third study generated identically to study 1. We found that the performance of our methods only improved for this additional data, see the supplement for further details and results.

### Application study

#### Study population

We used individual-level data from CECILE study [32], a French population-based case-control study on breast cancer (1,125 cases and 1,172 controls) and from the french case-control studies included in the EPITHYR consortium on thyroid cancer (CATHY, Young-thyr, and E3N studies totalling 1,129 women cases and 1,174 women controls) [33]. Only women of European ancestry were kept for the analyses.

Participants from CECILE study were genotyped using a customized microarray including variants from 28 candidate pathways (648 genes) selected from KEGG database and from a literature review. A total of 8,716 SNPs were selected to capture SNPs within 5 kb of each gene (pairwise approach with r2 > 0.8) with a minimum minor allele frequency (MAF) of 0.05 in the Caucasian population (CEU) genotyped by the HapMap Project (Data Release 21/Phase II, NCBI Build 36.1, assembly dbSNPb126) [32]. In EPITHYR, all subjects were genotyped using the Infinium OncoArray-500K BeadChip (Illumina). This array includes a genome-wide backbone of about 250,000 tag SNPs designed such that the large majority of common variants could be accurately imputed. Additional SNPs included dense coverage across known loci associated with common cancers, including breast cancer but not thyroid cancer. We added 13,759 custom markers of possible interest for thyroid cancer to the design of the chip [33]. Imputation of data from EPITHYR were then performed using the 1000 Genomes Project dataset as the reference panel (release of October 2014, version 3). Rare SNPs (MAF < 0.01) and palindromic SNPs were excluded. After quality controls (QC), we retained 6,677 SNPs available for both cancers.

As our approaches do not deal with overlapping groups, 10 non-overlapping candidate pathway were selected and only the SNPs related to those pathways were kept in the final datasets. Within each genes, SNPs were pruned for high pairwise correlation (r^2^ > 0.98). Then, only SNPs belonging to non overlapping groups (genes and pathways) were selected. At the end of the QC, the two datasets included the same panel of 3,766 SNPs within 331 genes and 10 pathways (see Table 1).

**Table 1:** Non-overlapping pathway chosen for the study

Pathway	Description	#Gene	#SNP
F _*obesity*	Obesity and obesity-related phenotypes	48	857
F _*DNA*	DNA repair	88	610
F _*circadian*	Circadian Rhythm	23	559
F _*xeno*	Xenobiotics metabolism	68	531
F _*pub*_*he*2010_4	Precocious or delayed puberty	16	329
F _*cell*_*cycle*	Cell cycle	19	249
F _*tobacco*_*hsa*00760	Nicotinate and nicotinamide metabolism	23	229
F _*inflammatory*	Inflammatory response	26	182
F _*oglyc*_*hsa*00511	Other glycan degradation	15	111
F _*folate*	Folate metabolism	5	50

#### Statistical analysis

We applied the three proposed methods to the investigation of pleiotropy between breast and thyroid cancers. The GMT and SGMT methods were both applied twice in order to consider both gene and pathway as different group structures. First, the tuning parameters has been calibrated using 5-fold cross-validation procedures. For SMT and GMT, we then performed the analysis using the values of *λ* parameter minimising the mean of the binomial deviance over 5 repetitions. For SGMT, the best couple of tuning parameter (*α*,*λ*) has been calibrated using 5 repetitions of 5-fold cross-validation. Once the methods were fit to the data, we explored the stability of the penalised methods using a bootstrap sampling strategy. We evaluated the methods on 10,000 bootstrap samples of the data using the tuning parameters from the original fit to the full dataset. We evaluated the frequency of selected SNPs (or groups for GMT) on the 10,000 bootstrap samples. Finally, we selected only the variables with a higher bootstrap selection rate than the non-selected variables from the original fit to the full dataset. For details see the supplementary material.

We also analysed these datasets using ASSET for an empirical comparison of the methods. We first performed GWAS analyses for breast and thyroid cancers separately in order to get summary statistics. As ASSET is based on p-values, we applied a FDR to correct for multiple testing. As we only have interest in identification of pleiotropic effects, we only considered SNPs detected in both datasets.

Furthermore, we compared the results of our proposed methods with previously published results on the same data using Bayesian meta-analysis models called GCPBayes at gene-level [34] which are based on summary statistics.

## Results

### Simulation results

Table 2 shows the estimated variable selection performance of the regression coefficients from the different penalised multi-task methods and the competitor ASSET. Selection performance was measured by the number of correctly selected variables, the true positive rate (TPR), the number of correctly non-selected zero variables, true negative rate (TNR) and the Mathew’s correlation coefficient (MCC). For ASSET, variables were said to be selected if they had a false discovery adjusted p-value lower than 0.05 at a variable-level. Groups were selected if the minimum adjusted p-value at a variable-level within the group was significant. We applied a strict bootstrapping approach for detecting effects using the penalised approaches. Specifically, for each simulated dataset we re-sampled the data 200 times and re-ran each of the penalised methods (SMT, GMT, SGMT and SGST) with their one-standard-error cross-validated *λ* and *α* values. We defined a variable to be selected (*active*) only if it was selected in every bootstrapped run.

**Table 2:** Average variable selection performance averaged across 100 simulated datasets with standard deviations in brackets

		Individual			Group		
	Method	MCC	TPR	TNR	MCC	TPR	TNR
Sim 1	SMT	0.29 (0.36)	0.22 (0.27)	1.00 (0.00)	0.41 (0.49)	0.41 (0.49)	1.00 (0.00)
	GMT	0.10 (0.14)	0.36 (0.48)	0.91 (0.11)	0.35 (0.49)	0.36 (0.48)	0.99 (0.05)
	SGMT	0.47 (0.39)	0.41 (0.37)	1.00 (0.00)	0.60 (0.49)	0.63 (0.49)	0.97 (0.09)
	ASSET	0.21 (0.34)	0.16 (0.26)	1.00 (0.00)	0.28 (0.45)	0.28 (0.45)	1.00 (0.00)
	GPA	0.03 (0.12)	0.09 (0.18)	0.93 (0.17)	0.04 (0.18)	0.21 (0.41)	0.83 (0.37)
	SGST	0.09 (0.21)	0.05 (0.12)	1 (0.00)	0.17 (0.38)	0.17 (0.38)	1 (0.03)
Sim 2	SMT	0.55 (0.15)	0.34 (0.16)	1.00 (0.00)	0.88 (0.20)	0.84 (0.25)	0.99 (0.03)
	GMT	0.34 (0.08)	0.80 (0.27)	0.83 (0.06)	0.85 (0.21)	0.80 (0.27)	1.00 (0.02)
	SGMT	0.72 (0.14)	0.56 (0.20)	1.00 (0.00)	0.95 (0.11)	0.95 (0.15)	0.99 (0.04)
	ASSET	0.46 (0.19)	0.26 (0.15)	1.00 (0.00)	0.74 (0.30)	0.70 (0.33)	0.99 (0.04)
	GPA	0.22 (0.2)	0.11 (0.12)	0.99 (0.07)	0.43 (0.39)	0.39 (0.37)	0.98 (0.14)
	SGST	0.01 (0.05)	0.00 (0.02)	1 (0.00)	0.03 (0.13)	0.02 (0.1)	1.00 (0.00)
Sim 3	SMT	0.46 (0.08)	0.24 (0.08)	1.00 (0.00)	0.91 (0.12)	0.89 (0.16)	0.99 (0.03)
	GMT	0.57 (0.03)	0.98 (0.06)	0.84 (0.01)	0.99 (0.05)	0.98 (0.06)	1.00 (0.01)
	SGMT	0.73 (0.09)	0.59 (0.13)	1.00 (0.00)	0.97 (0.07)	0.99 (0.04)	0.99 (0.03)
	ASSET	0.33 (0.11)	0.14 (0.08)	1.00 (0.00)	0.77 (0.20)	0.71 (0.26)	0.99 (0.04)
	GPA	0.23 (0.09)	0.07 (0.04)	1.00 (0.00)	0.61 (0.23)	0.49 (0.26)	1.00 (0.01)
	SGST	0.00 (0.00)	0.00 (0.00)	1 (0.00)	0.00 (0.00)	0.00 (0.00)	1.00 (0.00)
Sim 4	SMT	0.21 (0.05)	0.06 (0.02)	1.00 (0.00)	0.70 (0.15)	0.61 (0.17)	0.99 (0.02)
	GMT	0.86 (0.02)	1.00 (0.03)	0.94 (0.00)	1.00 (0.02)	1.00 (0.03)	1.00 (0.00)
	SGMT	0.56 (0.04)	0.38 (0.04)	1.00 (0.00)	0.99 (0.02)	1.00 (0.02)	1.00 (0.01)
	ASSET	0.13 (0.08)	0.03 (0.03)	1.00 (0.00)	0.46 (0.25)	0.34 (0.24)	0.99 (0.02)
	GPA	0.11 (0.05)	0.02 (0.01)	1.00 (0.00)	0.43 (0.21)	0.28 (0.18)	1.00 (0.01)
	SGST	0.00 (0.00)	0.00 (0.00)	1.00 (0.00)	0.00 (0.00)	0.00 (0.00)	1.00 (0.00)

Measures of performance are based on variable (pleiotropic) effect recovery and group effect recovery

This differs from the bootstrapping approach in the application which promotes a higher sensitivity as it is more suitable in genomic context in order to detect more potential signals. We report results using the bootstrapping from the applicaiton in the supplementary material. Our results here gives a more comparable control of the false discovery rate with the ASSET and GPA approaches, and thus a fairer comparison of the methods. Results comparing the methods using the bootstrapping selection approach from the application are given in the supplementary material. Table 2 shows the performance of our methods. We have given the performance at both variable pleiotropic signal detection level (variable-level) and at their effect detection level for groups of variables (group-level).

A consequence of using this strict FDR and bootstrapping procedure is that the true negative rate is almost consistently at 100% with low standard error. The MCC and TPR in comparison have higher variability and differ more amongst the methods. We note also that the variability of the methods appears to decrease for the larger simulations. This is because the smaller simulation settings have a small number of true variables and consequently there is more variance in the estimate of the true positive ratio. For example Sim 1 has only 2 active variables so there are 3 possible TPR values for any dataset.

The simulations results in Table 2 show clearly that single-task method is not efficient at detecting pleiotropic effects. For every scenario the method struggles to find signal in the data. Regarding methods designed for pleiotropy, GPA was outperformed by every other methods at both variable and group level. Both ASSET and SMT have comparable performance in detecting effects at a *variable-level* with SMT having slightly better performance in TPR and MCC. In simulation 4 where group information is most relevant SMT and ASSET suffer in terms of TPR, indicating that the method struggles to detect true effects. Meanwhile GMT has the best performance for simulation 4. However, for simulation 1 and 2, GMT has lower performances in term of MCC compared to SMT and SGMT, although GMT is still good in term of TPR. In simulation 1, 2 and 3, SGMT also shows the best performances in MCC overall. In simulation 4, SGMT is outperformed by GMT in MCC, but still shows better performances than SMT and ASSET. SGMT offers the best compromise overall at variable-level.

At a group-level, the Multi-Task methods all had significantly better performance than the ASSET approach for TRP, TNR and MCC. Methods which incorporated grouping information were even better yet in their accuracy for recovering the active groups of variables. Moreover, we found that the SGMT method was able to outperform the GMT method for selection at a group-level when there was sparsity within the active groups.

Finally, in Table 3 we comment on the reconstruction error for the different methods designed for pleiotropy detection. The estimated regression coefficients for ASSET and GPA were then taken to be the summary statistic OLS estimate for the selected variables and zero elsewhere. For the Multi-Task approaches the reconstruction error was taken using the estimate corresponding to the one-standard error rule from a run of 10-fold cross-validation. The GPA method performs poorly in reconstruction compared to other approaches. In general ASSET performs poorly in reconstruction compared to the penalised approaches. This difference becomes more apparent for problems with high dimension where the reconstruction challenge is harder. Again we see similarity between ASSET and SMT, with SMT having slightly better performance. Over all simulation settings SGMT appears to be competitive or attain the best L1 or L2 reconstruction error.

**Table 3:** Average reconstruction error for the different methods over 100 simulated datasets with standard deviations in brackets

	L1				L2			
Method	Sim 1	Sim 2	Sim 3	Sim 4	Sim 1	Sim 2	Sim 3	Sim 4
SMT	2.76 (0.94)	9.62 (1.16)	41.95 (2.33)	193.14 (2.64)	1.20 (0.27)	2.22 (0.29)	4.95 (0.29)	11.80 (0.18)
GMT	3.67 (0.64)	13.60 (0.58)	45.68 (1.35)	174.58 (3.04)	1.52 (0.11)	2.67 (0.18)	5.01 (0.21)	10.69 (0.21)
SGMT	3.66 (2.05)	10.05 (1.57)	39.63 (2.30)	176.60 (3.64)	1.21 (0.31)	2.09 (0.25)	4.52 (0.27)	10.85 (0.24)
ASSET	2.88 (0.55)	10.25 (1.48)	47.36 (1.99)	202.60 (1.87)	1.47 (0.23)	2.79 (0.26)	6.04 (0.19)	12.69 (0.1)
GPA	6.34 (7.45)	12.33 (3.67)	49.04 (1.27)	203.32 (1.13)	1.87 (0.64)	3.06 (0.19)	6.21 (0.11)	12.73 (0.06)

The estimated coefficients for the penalised methods correspond to the estimate with tuning parameters chosen from cross validation. The estimated coefficients for ASSET and GPA are set using the summary statistics of the active variables. An active variable for ASSET and GPA was one with a FDR corrected p-value less than 0.05

### Application results

We first run the analyses at SNP-level i.e. that do not take into account for group structure. As a results, no significant SNP was detected by ASSET after correction for multiple testing. Our proposed SMT method selected 11 SNPs from which 8 have been confirmed by the bootstrap sampling strategy. The results of the analyses with the proposed methods are shown in Table 4.

**Table 4:** Pleiotropic SNPs selected by our different approaches. For each method, we reported if the SNP effect was find in the same direction between the two studies (+), the opposite direction (-) or not selected (ns)

SNP	Chr	Pos (kbp)	EA	BA	DE		SGMT	
					BC	TC	Gene	Pathway
rs1342862 *	1	72,657	G	A	−	−	NEGR1	F _*obesity*
rs17483835 *	1	183,297	A	G	−	−		F _*tobacco*_*hsa*00760
rs17332991 *	5	60,179	A	C	−	−	ERCC8	F _*DNA*
rs6151640 *	5	79,967	G	C	−	−		F _*DNA*
rs249634 *	5	80,164	G	A	+	−		F _*DNA*
rs4978820 *	9	110,057	A	G	−	−		F _*DNA*
rs4255624	12	24,960	G	A	−	−		F _*pub*_*he*2010_4
rs878156 *	14	20,824	G	A	−	+	PARP2	F _*DNA*
rs1482057 *	15	61,064	A	C	−	+	RORA **	F _*circadian*
rs12150110	17	11,962	A	G	+	+		F _*cell*_*cycle*
rs3087592	22	41,079	A	G	+	−		F _*obesity*

Chr: chromosome; EA: effect allele; BA: baseline allele; DE: direction of effects; BC: breast cancer; TC: thyroid cancer; * SNP selected by SMT; ** Gene selected by GMT

**Fig. 2:**
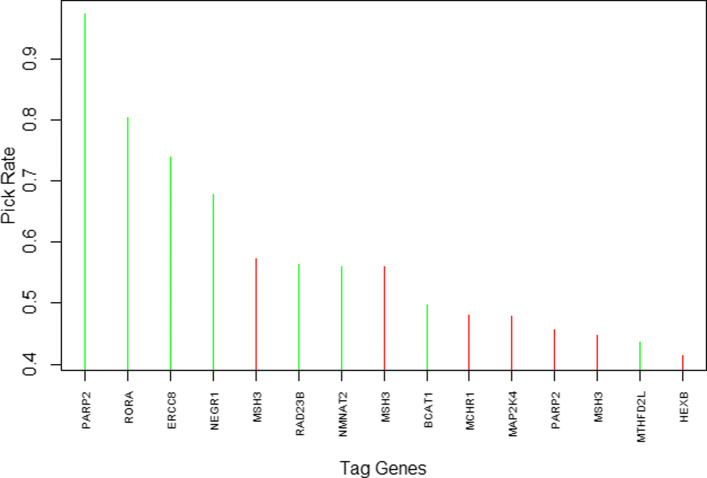
First 15 selected SNPs in the bootstrapped analysis with gene as group structure, ordered by frequency of appearance. The name of corresponding genes are mentioned. The 8 signals selected in the analysis on real datasets are represented in green

We then performed gene-level analysis using GMT and SGMT. GMT selected the gene *RORA* (retinoic acid receptor-related orphan receptor alpha) as pleiotropic. This gene located on the chromosome 15 is involved in the regulation of circadian rhythms. *RORA* was still selected after the bootstrap procedure. However, GMT does not perform variable selection for variables within a group. SGMT selected *RORA* and a further seven genes. After the bootstrap procedure, only 4 SNPs remained selected, each located in intron of a different tag gene: rs1482057 in *RORA*, rs1342862 in *NEGR1* (neuronal growth regulator 1), rs17332991 in *ERCC8* (excision repair 8, CSA ubiquitin ligase complex subunit), and rs878156 in *PARP2* (poly(ADP-ribose) polymerase 2). These SNPs were also selected by SMT. *NEGR1* located in chromosome 1 is an obesity-related gene. *PARP2* located in chromosome 14 encodes for a class of nuclear enzymes involved in the pathogenesis of diverse gynecologic tumors [35]. The frequency of the most selected SNPs and the corresponding tag genes are shown in Fig. 2.

**Fig. 3:**
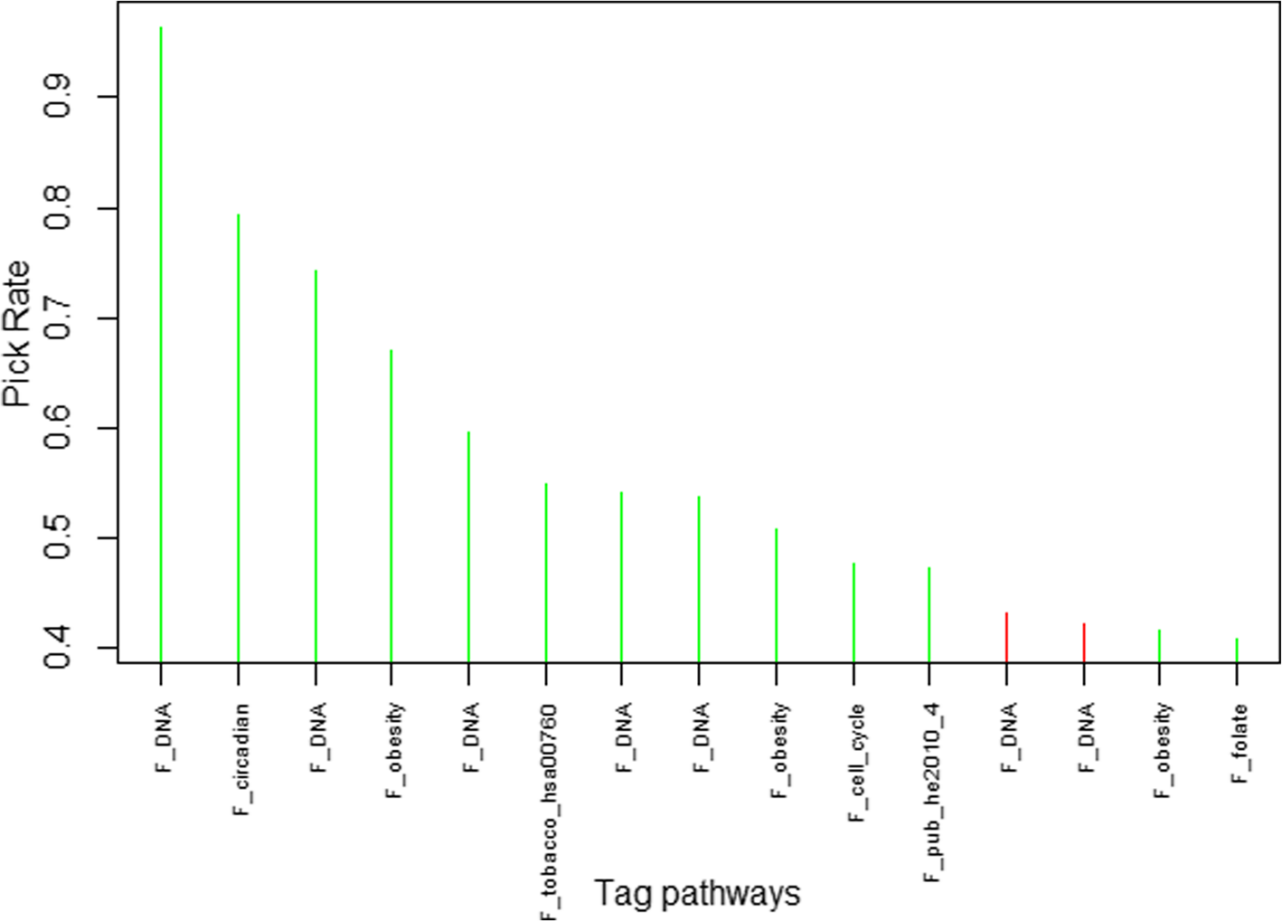
First 15 selected SNPs in the bootstrapped analysis with pathway as group structure, ordered by frequency of appearance. The name of corresponding pathways are mentioned. The 13 signals selected in the analysis on real datasets are represented in green

Analysis with pathway as group structure using GMT did not allow any pathway detection. However, SGMT with pathway as the grouping structure detected 13 consistently selected signals (see Fig. 3). The bootstrap sampling analysis revealed consistent results, as 11 out of 13 SNPs were the most frequently selected SNPs with analyses on bootstrapped samples. The final 11 pleiotropic hits selected by SGMT belonged to 6 different pathways. To note, SGMT allowed to detect the 8 SNPs that were already selected by SMT, but also allow to detect 3 new signals by considering the pathway structure (see Table 4), with one additional pleiotropic signal in the F _*obesity* pathway and two single signals in the F _*pub*_*he*2010_4 and F _*cell*_*cycle* pathways.

It can be highlighted that 2 out of 4 selected genes by SGMT were selected by a previous analyse on the same data using GCPBayes Bayesian meta-analysis methods at gene-level [34]: *RORA* and *NEGR1*. Again, the gene *PARP2* appeared to a suggestive threshold as a gene with potential pleiotropic effect which could have been selected with a larger sample size. The fourth gene, *ERCC8*, was not selected by GCBPayes. Also, GCPBayes selected 3 different SNPs as specific effects in *NEGR1* but not rs1342862, that is not in linkage disequilibrium with these 3 SNPs. However, rs1342862 has a D’=1 with rs12133119 and rs17588812, indicating these SNPs share co-inherited alleles. No specific SNP corresponding to *RORA* was selected by GCPBayes.

## Discussion

The proposed methods can bring power to detect new shared genetic effects between multiple diseases by allowing to simultaneously analyse multiple variables and traits. This allow us to take into account for the correlations between variables and between traits in the analysis, in contrary to methods based on summary statistics from GWAS. Also, our methods allow for incorporating prior knowledge such as group structure corresponding to genes or pathways which can increase the statistical power to identify important risk variants. However, it should be noted that taking into account the group structure requires labeling the data without overlap between the groups, which may require re-partitioning the variables within groups if variables are linked to several groups.

A simulation study showed excellent performances of our proposed methods. Even without incorporating grouping knowledge, our SMT method outperformed ASSET in almost all situations. GMT, which only considered variable selection at a group level, showed great performance. This was especially clear when the ratio of nonzero to zero variables within a group was high. We note that the correlation structure of genetic data that is the LD, should help GMT to perform reasonably well even when the ratio of true pleiotropic variables in a group is lower, what is likely in real data. More generally, higher correlation in the data would be in favour of multivariates methods such as our proposed methods. The SGMT method that allow selection at variable and group-level takes the best parts of both SMT and GMT. This method showed the highest performances in almost all simulations and was comparable to the best performance at both variable-level and group-levels.

The proposed approaches were applied to the investigation of the shared genetic effects between thyroid and breast cancers in candidate pathways. The application study have shown our proposed methods are capable of detecting new signals would not be detected by ASSET. All the multi-task methods were applied to both genes and pathways as group structure. The SGMT method allowed to detect more signals than SMT and GMT methods. SGMT detected 11 pleiotropics SNPs in 6 different pathways, from which 8 SNPs were also detected by SMT. The analysis with genes as group structure highlighted 4 out of these 11 selected variables located in 4 different genes, from which *RORA* was detected by GMT leading to strong evidence about implication of this gene in the mechanism of both cancers. Interestingly, *RORA* is part of the core circardian genes and variants in these gene were previously reported to be associated to several cancers, including breast, prostate and pancreatic cancers [36]. This gene is suspected to play a role in tumor suppression and was found to be inactivated in multiple cancers [37].

## Conclusion

We present three novel feature selection methods at group and variable level adapted for pleiotropy detection in GWAS data using the multi-task regression framework. These methods use penalised likelihood methods, exploiting different penalties, to induce structured sparsity at a group and SNP level. Our methods are developed to model pleiotropic correlation amongst jointly analysed traits and account for the effect of linkage disequilibrium by incorporating known group structures such as gene or pathway. They take into account heterogeneity in the size and direction of the genetic effects across traits. An ADMM algorithm is used to solve the penalised regression problems. We have conducted simulation studies to evaluate the performance of our method compared to one of the most popular method adapted for pleiotropy for practitioners. We have applied our methods to the analysis of two datasets on breast and thyroid cancers.

Future work could consider extending these methods to allow for groups with overlap with extensions to the ADMM optimisation or alternative efficient methods [38]. Other extensions could include generalising the approach for the joint analysis of multiple generalised likelihoods (e.g. logistic, linear, Poisson, etc). Further investigation of the choice of weights in the adaptive component of the penalisation could also be of theoretical and practical interest. Another future avenue of research would be development of p-value calculations and more technical FDR control measures in line with the theoretical development of Lockhart et al. [39] or Candés et al. [40]. In conclusion, the proposed multi-task regression methods were seen to be more powerful than methods based on summary statistics to detect new pleiotropic effects in complex diseases, and are computationally feasible. These methods allow us to take into prior knowledge in the analysis of the genetic data as the biological structures of genes or pathways, and hence it allow to select important risk variants or group structures with more biological meaning. These methods are likely to be of interest for other application to detect non-zero effects of possible different directions in structured data. The methods have been implemented in a user-friendly R statistical package called “SGMT”, available at https://github.com/matt-sutton/SGMT.

## Data Availability

Methods in a usable R package and simulated data are available at https://github.com/matt-sutton/SGMT. Access to individual data are legally restricted due to privacy and ethical restrictions. Access to the data would require to sign a Data Transfer Agreement with INSERM, which insures that the data can be used only for the purpose of research in accordance with the IRB-approved protocol and patient consent form. The point of contact for data access is Therese Truong (therese.truong@inserm.fr)

## Abbreviations

SMT: Sparse multi-task
GMT: Group multi-task
SGMT: Sparse-group multi-task
GWAS: Genome-wide association study
SNP: Single nucleotide polymorphism
LASSO: Least absolute shrinkage and selection operator
ADMM: Alternating direction method of multipliers
L-BFGS: Limited-memory Brouden-Fletcher-Golfarb-Shannon algorithm
OLS: Ordinary least squares
ASSET: association analysis based on subsets
RORA: retinoic acid receptor-related orphan receptor alpha
ERCC: excision repair
PARP: Poly(ADP-robose) polymerase
NEGR: Neuronal growth regulator
LD: Linkage-disequilibrium
